# Intense Exercise Promotes Adult Hippocampal Neurogenesis But Not Spatial Discrimination

**DOI:** 10.3389/fncel.2017.00013

**Published:** 2017-01-31

**Authors:** Ji H. So, Chao Huang, Minyan Ge, Guangyao Cai, Lanqiu Zhang, Yisheng Lu, Yangling Mu

**Affiliations:** ^1^Department of Physiology, School of Basic Medicine, Huazhong University of Science and Technology Wuhan, China; ^2^Tianjin Institute of Integrative Medicine for Acute Abdominal Disease, Nankai Hospital Tianjin, China; ^3^Institute of Brain Research, Collaborative Innovation Center for Brain Science, Huazhong University of Science and Technology Wuhan, China; ^4^Hubei Key Laboratory of Drug Target Research and Pharmacodynamic Evaluation, Huazhong University of Science and Technology Wuhan, China

**Keywords:** hippocampus, pattern separation, neurotrophic factors, erythropoietin, prohibitin

## Abstract

Hippocampal neurogenesis persists throughout adult life and plays an important role in learning and memory. Although the influence of physical exercise on neurogenesis has been intensively studied, there is controversy in regard to how the impact of exercise may vary with its regime. Less is known about how distinct exercise paradigms may differentially affect the learning behavior. Here we found that, chronic moderate treadmill running led to an increase of cell proliferation, survival, neuronal differentiation, and migration. In contrast, intense running only promoted neuronal differentiation and migration, which was accompanied with lower expressions of vascular endothelial growth factor, brain-derived neurotrophic factor, insulin-like growth factor 1, and erythropoietin. In addition, the intensely but not mildly exercised animals exhibited a lower mitochondrial activity in the dentate gyrus. Correspondingly, neurogenesis induced by moderate but not intense exercise was sufficient to improve the animal’s ability in spatial pattern separation. Our data indicate that the effect of exercise on spatial learning is intensity-dependent and may involve mechanisms other than a simple increase in the number of new neurons.

## Introduction

New neurons are continuously produced in the dentate gyrus (DG) of adult animals (Altman and Das, 1965; Kaplan and Hinds, 1977; Zhao et al., 2008). Although the exact function of adult neurogenesis remains an open question, accumulating evidence suggests that nascent dentate granule cells (DGCs) play an important role in memory formation, consolidation and retrieval (Kempermann et al., 1997; Shors et al., 2001; Kitamura et al., 2009; Gu et al., 2012). The addition of new DGCs has also been implicated in mood regulation (Santarelli et al., 2003). In contrast, decline in neurogenesis is linked to various mental disorders, such as Alzheimer’s disease (Mu and Gage, 2011), schizophrenia, major depression, and post-traumatic stress disorder (Christian et al., 2014). It is thus necessary to elucidate factors that modulate adult hippocampal neurogenesis.

The adult-born DGCs originate from neural stem cells (NSCs) located in the subgranular zone. Before growing into functionally mature neurons, they go through a development process mainly consisting of cell proliferation, neuronal differentiation and synaptic integration. Each stage can be influenced by numerous physiological and pathological factors (Mu et al., 2010). A large body of literature has reported benefits of physical exercise on neurogenesis in the adult DG (van Praag et al., 1999; Vivar et al., 2013). Specifically, running can enhance cell proliferation by shortening the cell cycle length of NSCs (Farioli-Vecchioli et al., 2014). It can also promote functional maturation and prevent the death of new neurons (Ernst et al., 2006; Snyder et al., 2009). Since exercise increases blood flow and thus delivery of oxygen and nutrients to the brain, it is commonly considered to exert neurogenic effects through altering the biochemical microenvironment of adult NSCs. Many growth factors and neurotransmitters, such as brain-derived neurotrophic factor (BDNF), insulin-like growth factor 1 (IGF-1), and serotonin, increase with running and have been causally linked to running-induced upregulation of neurogenesis (Trejo et al., 2001; Radley and Jacobs, 2002; Ernst et al., 2006; Vivar et al., 2013). Behaviorally, enhanced neurogenesis following physical exercise is thought to underlie the improvement in a variety of hippocampus-dependent learning tasks (van Praag et al., 1999; Creer et al., 2010), and may even contribute to amelioration of depression and cognitive deficits associated with several neurodegenerative disorders (Xu et al., 2010; Krogh et al., 2011).

In spite of these findings, the impact of exercise on adult neurogenesis may vary with factors, such as the training apparatus, duration, and intensity. For example, voluntary and forced exercise have different effects on hippocampal BDNF levels (Uysal et al., 2015). Short-term exercise usually potentiates cell proliferation, while long-term exercise primarily enhances neuronal differentiation and survival (Kim et al., 2003; Bednarczyk et al., 2009). Low- to moderate-intensity, but not high-intensity exercise can benefit adult hippocampal neurogenesis, upregulate the expression of several related molecules and improve spatial learning (Inoue et al., 2015). It should be noted that, although exercise itself often results in activation of the hypothalamus-pituitary-adrenal (HPA) axis and subsequent production of stress hormones, forced exercise usually involves the use of a punishment that causes extra stress to the animals, in particular to the heavy exercisers. Since mild stress can increase adult neurogenesis, while prolonged and unpredictable stress has an opposite effect (Mirescu and Gould, 2006), it is possible that high stress response linked to punishment masks the benefits of high-intensity training. In the current study, we built animals models of mild and intense long-term exercise without introducing differences in their stress levels, which warranted comparisons between them to examine solely the effects of distinct exercise intensities on neurogenesis and the associated molecular mechanisms. In addition, given the role of neurogenesis in pattern separation, we aimed to investigate the relation between exercise intensity and the animal’s ability in spatial discrimination.

## Materials and Methods

### Subjects

C57BL/6 female mice (8 weeks old) used in this study were purchased from the Experimental Animals Center of Tongji Medical College, Huazhong University of Science and Technology. Animals were housed in polycarbonate cages (five mice per cage) on a 12-h light/dark schedule (light on at 7:00 a.m.), with free access to food and water unless specified. Estrous stage was evaluated as previously described (Lagace et al., 2007). Briefly, vaginal smears were taken from 14-week-old mice. The cells were spreaded on slides and compared with standard hematoxylin and eosin (H&E) staining (Wuhan Goodbio Technology, China). The experimental protocols were carried out in accordance with the Committee of Animal Care of Huazhong University of Science and Technology.

### Exercise Training

The animals were randomly separated into three groups: sedentary control (CT), moderate exercise (ME), and fatiguing exercise (FE). The exercise apparatus is a six-lane treadmill placed at an inclination of 0° (Columbus Instruments, China). After acclimation (40-min running at a speed of 10 m/min for 3 days), the ME and FE groups were subjected to daily 40-min treadmill exercise for 6 weeks. The ME mice ran at 10 m/min for the first 5 min, and at 15 m/min for the remaining 35 min. The FE mice ran at 10 m/min for the first 5 min, and then the speed was increased by 5 m/min every 1 min, until it reached 30 m/min. A gentle tap on the tail with a soft bristle brush was used to encourage mice to keep running on the treadmill. In rare cases, the animals were excluded from this study if they refused to run. The CT mice did not receive any exercise training and spent their entire time in their home cages. Physical conditions and body weights of all the animals were monitored throughout the training period.

### Lactate and Cortisol Measurements

Serum samples were prepared from tail vein blood immediately after the mice finished the last running session, by centrifugation at 3000 × *g* for 5 min at room temperature. Lactate and cortisol levels in serum were determined using a lactate assay kit (Nanjing Jiancheng Bioengineering Institute) and a cortisol enzyme-linked immunosorbent assay (ELISA) kit (R&D Systems) according to the manufacturers’ protocols, respectively. The optical density of each well was measured at 530 nm for lactate and at 450 nm for cortisol using microplate reader (TECAN Austria GmbH 5082 Grodig, Austria). The concentrations were calculated by comparing samples to the standard curves generated with the kits. All the assays were conducted within 3 h after the serum samples were obtained.

### Protein Assays

The hippocampus of each hemisphere was first dissected out. The hippocampi and the remaining brain tissue were then separately homogenized (Glas-Col homogenizer) in ice-cold lysis buffer containing 100 mM PIPES (pH 7.0), 500 mM NaCl, 0.2% Triton X-100, 0.1% NaN_3_, 2% BSA, 2 mM EDTA⋅Na_2_⋅2H_2_O, 200 μM PMSF, 10 μM leupeptin, 0.3 μM aprotinin, and 1 μM pepstatin. The homogenates were centrifuged for 30 min at 16,000 × *g* at 4°C. The supernatants were aliquoted and stored at -80°C until the time of assay. The contents of hippocampal vascular endothelial growth factor (VEGF), BDNF, EPO, and brain IGF-1 were quantified using ELISA Kits, complying with the manufacturers’ instructions. Briefly, a specific monoclonal or polyclonal antibody was pre-coated onto a microplate. Standards, control, and diluted samples were pipetted into the wells. After washing, the captured molecules bound the second specific polyclonal antibody. The amount was detected by an enzyme-substrate reaction that yielded color change. The color intensity was measured using microplate reader (TECAN Austria GmbH 5082 Grodig, Austria) and the sample values were then read off the standard curve. All kits were purchased from R&D Systems (Minneapolis, MN, USA), except that the BDNF Emax immunoassay system was from Promega Co (Madison, WI, USA).

### BrdU Injections

Mice received an intraperitoneal injection of BrdU (50 μg/g body weight) during the 2nd week of treadmill running. A sterile solution of 10 mg/ml BrdU (Sigma) in 0.9% NaCl was used. Injections were given twice a day with a 4 h interval for six consecutive days. Animals were sacrificed the day after the last BrdU injection to assess cell proliferation. To evaluate cell survival, animals were sacrificed immediately after the 6-week training session was finished.

### Immunohistochemistry

The mice were anesthetized with chloral hydrate and perfused with phosphate buffered saline (PBS) and 4% paraformaldehyde (PFA) in sequence. The brains were post-fixed in 4% PFA, equilibrated in 30% sucrose and serially sectioned in the coronal plane at 40 μm with a cryotome (SLEE, Mainz, Germany). Immunofluorescent double-labeling was performed as previously described (Kuhn et al., 1996). For BrdU-NeuN double staining, NeuN was first labeled with a rabbit monoclonal antibody (Abcam; diluted 1:1000) and visualized with an Alexa Fluor 488-conjugated secondary goat anti-rabbit antibody (Jackson ImmunoResearch Laboratories; diluted 1:500). After being fixed and rinsed, sections were incubated with a mouse monoclonal antibody against BrdU (Millipore; diluted 1:200) followed by a Cy3-conjugated secondary goat anti-mouse antibody (Jackson ImmunoResearch Laboratories; diluted 1:500). For Ki67-NeuN double staining, Ki67 was detected with a rabbit monoclonal antibody (Abcam; diluted 1:1000) and visualized with an Alexa Fluor 488-coupled secondary goat anti-rabbit antibody (Jackson ImmunoResearch Laboratories; diluted 1:500). NeuN was detected with a mouse monoclonal antibody (Millipore; diluted 1:200) and visualized with a Cy3-coupled secondary goat anti-mouse antibody (Jackson ImmunoResearch Laboratories; diluted 1:500). For prohibitin staining, a rabbit antibody (Santa Cruz; diluted 1:300) and an FITC-conjugated secondary goat anti-rabbit antibody (Earthox; diluted 1:150) were used.

### Cell Counts

Brain sections were mounted on glass slides and fluorescence signals were imaged using a Zeiss LSM710 confocal laser scanning microscope. Sequential scanning was performed with a 1 μm separation along the *z*-axis. Image stacks were compressed into a single plane using ImageJ2x software with an average intensity projection. Quantification of labeling was determined by counting all fluorescent cells in every sixth coronal section. To determine the distribution of newborn cells, the granule cell layer (GCL) was divided into three layers in a manner similar to that previously described (Mathews et al., 2010). Briefly, the GCL borders were first determined by NeuN-labeling. The GCL width was determined using the gridlines. The GCL was then partitioned into three layers of approximately equal thickness. The numbers of BrdU- or Ki67-positive cells in each layer were quantified.

### Radial Arm Maze (RAM) Task

The RAM testing was conducted in an eight-arm maze consisting of a central octagonal (48 cm across) and eight radially extending arms (15 cm wide × 45 cm long × 23 cm high) placed 65 cm above the floor. The maze was located in a room with many extra-maze visual cues. One week prior to the behavioral training, all animals were food restricted to reach ∼90% of their initial body weights. Following one day habituation, mice received training and testing over 15 consecutive days, four trials per day. Each trial consisted of a sample phase and a choice phase. During the sample phase, all arms except a start arm and the sample arm were blocked. When animals spent 10 s after eating up a small food pellet in the sample arm or returned to the center from the sample arm, they were removed from the maze. During the choice phase, the start arm, the sample arm (without food) and an additional arm baited with a food pellet were open. The arm containing reward were separated from the sample arm by 2, 3, or 4 arms. If mice entered the baited arm, they were regarded as making correct choices. Otherwise, they were allowed to self-correct and to consume the food before removal from the maze. The combinations of start, sample, and additional rewarded arms were randomized for each trial.

### Statistical Analysis

The results were presented as the mean ± standard error of the mean. One-way ANOVA followed by Tukey’s *post hoc* test was applied for analysis of differences between groups unless specified. Statistical differences were considered to be significant when *P* < 0.05.

## Results

### Animal Models of Exercise

**FIGURE 1:**
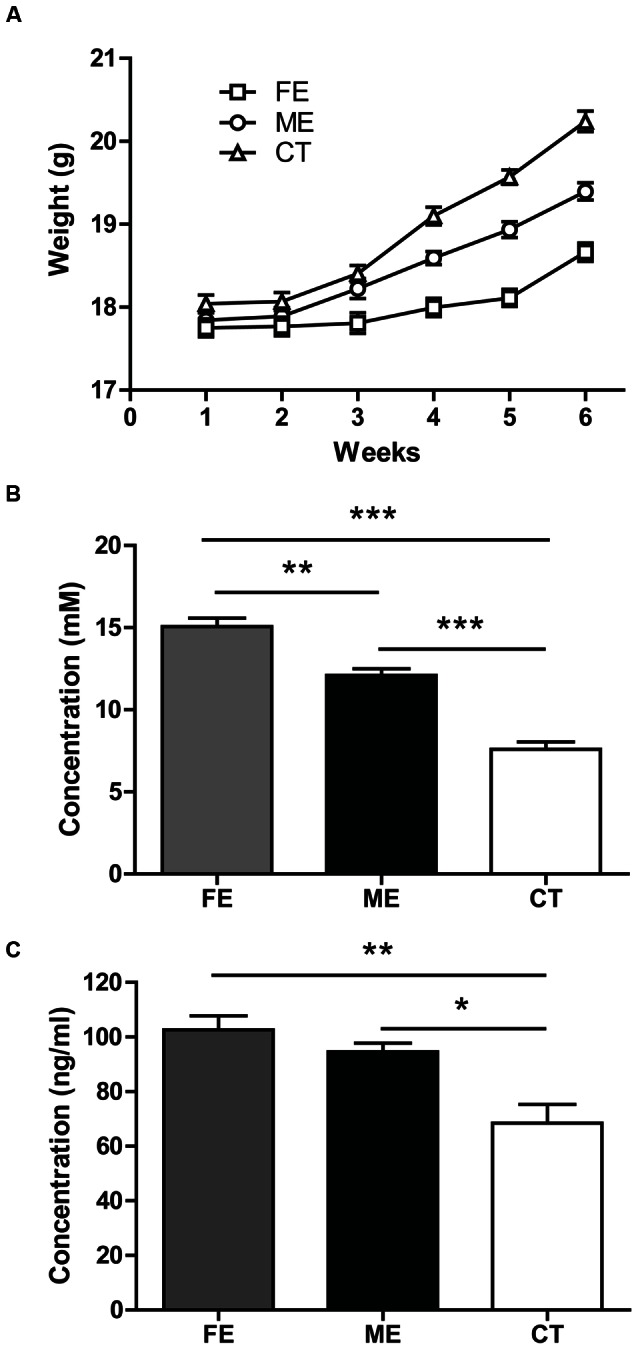
**Parameters indicating different exercise intensities. (A)** After 6 weeks of forced treadmill running, the fatiguing exercise (FE) group weighs less than the moderate exercise (ME) group, while the ME group weighs less than the control (CT) group. Two-way ANOVA, *F*_2,72_ = 37.69, *P <* 0.0001; *post hoc* test: ME vs. CT: *P <* 0.001; FE vs. CT: *P* < 0.0001; ME vs. FE: *P* < 0.0001; *n* = 25 in each group. **(B)** Following the last treadmill running session, blood lactate levels in FE mice are significantly higher than those in the ME mice, and blood lactate levels in ME mice are significantly higher than those in CT mice. One-way ANOVA, *F*_2,12_ = 68.40, *P* < 0.0001; *post hoc* test: ME vs. CT: ^∗∗∗^*P <* 0.001; FE vs. CT: ^∗∗∗^*P* < 0.001; ME vs. FE: ^∗∗^*P <* 0.01; *n* = 5 in each group. **(C)** Concentrations of serum cortisol in FE or ME mice are significantly higher than those in the CT mice. There is no significant difference between the FE and ME groups. One-way ANOVA, *F*_2,12_ = 11.52, *P* = 0.0016; *post hoc* test: ME vs. CT: ^∗^*P <* 0.05; FE vs. CT: ^∗∗^*P* < 0.01; FE vs. ME: *P* > 0.05; *n* = 5 in each group.

To assess the exercise intensity, we measured the body weight of each individual mouse every week at the same time. During the first 2 weeks of forced treadmill running, the average body weights of ME and FE mice were comparable to that of the controls (*n* = 25 in each group). All groups gained weights at the end of training (two-way ANOVA, *F*_5,360_ = 152.1 *P* < 0.0001; *post hoc* test: CT, ME, and FE: *P* < 0.0001, **Figure 1A**). However, the mean weight of FE mice was lower than that of ME or CT mice, and ME mice weighed significantly less than CT mice (FE: 18.66 ± 0.11 g; ME: 19.40 ± 0.10 g; CT: 20.24 ± 0.12 g; **Figure 1A**). We also measured the blood lactate concentrations in all the animals immediately following exercise. In comparison to CT mice, ME mice had a significantly higher level of lactate, and FE mice had even more lactate accumulation than the ME group (FE: 15.06 ± 0.51 mM; ME: 12.10 ± 0.40 mM; CT: 7.60 ± 0.43 mM; **Figure 1B**). These results suggest that our current animal models are suitable for studies on different exercise intensities.

Forced running usually involves the use of electrical shocks that may stress the animals (Inoue et al., 2015). To minimize the stress, we applied a gentle tap on the tail using a soft bristle brush instead to keep the mice running when necessary. Given that mouse serum cortisol and corticosterone are closely correlated in dynamics and cortisol is a more stable and quicker responder to stress (Gong et al., 2015), here we monitored the concentration of cortisol rather than corticosterone. The serum cortisol was found to be significantly higher in FE and ME mice than in the sedentary controls (FE: 102.6 ± 5.1 ng/ml; ME: 94.56 ± 3.19 ng/ml; CT: 68.42 ± 6.84 ng/ml; **Figure 1C**), but the FE group did not show a further cortisol increase as compared to the ME group. Thus, the exercise intensity effects could be investigated without confounding with the stress effects using our current animal models.

### Intensity-Dependent Effects of Exercise on Hippocampal Neurogenesis

**FIGURE 2:**
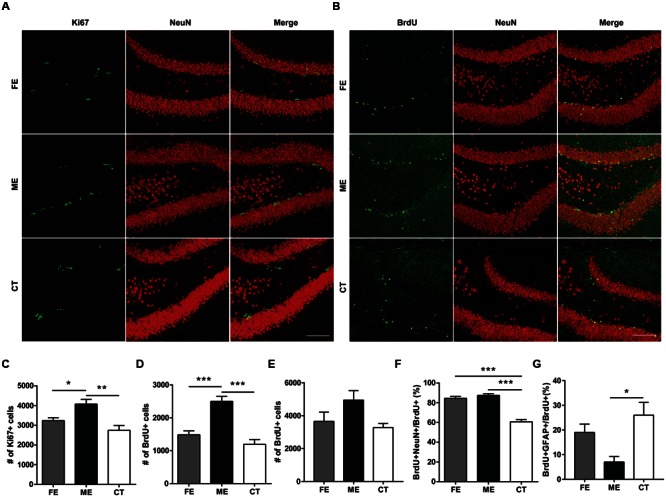
**Cellular proliferation, differentiation, and survival in the dentate gyrus (DG) of mice subjected to distinct training paradigms. (A)** Representative images of Ki67+ cells and their colocalization with NeuN in FE, ME, and CT mice, respectively. Scale bar = 100 μm. **(B)** Representative images of BrdU+ cells and their colocalization with NeuN in FE, ME, and CT mice, respectively. Scale bar = 100 μm. **(C)** Quantification of Ki67+ cells reveals a significant decrease in their total number in FE mice when compared to ME mice. One-way ANOVA, *F*_2,12_ = 9.429, *P* = 0.0035; *post hoc* test: ME vs. FE: ^∗^*P <* 0.05; ME vs. CT: ^∗∗^*P* < 0.01; FE vs. CT: *P* > 0.05; *n* = 5 in each group. **(D)** Quantification of BrdU+ cells reveals a significant decrease in their total number in FE mice as compared to ME mice. One-way ANOVA, *F*_2,12_ = 24.06, *P* < 0.0001; *post hoc* test: ME vs. FE: ^∗∗∗^*P <* 0.001; ME vs. CT: ^∗∗∗^*P <* 0.001; FE vs. CT: *P* > 0.05; *n* = 5 in each group. **(E)** Cellular proliferation in the DG of mice subjected to 2-week training. Quantification of BrdU+ cells reveals no significant change in their total number in FE or ME mice. One-way ANOVA, *F*_2,12_ = 3.254, *P* = 0.0743; *n* = 5 in each group. **(F)** Quantification of the ratios of BrdU+NeuN+ cells to BrdU+ cells in the FE, ME, and CT groups. One-way ANOVA, *F*_2,12_ = 54.26, *P* < 0.0001; *post hoc* test: ME vs. CT: ^∗∗∗^*P <* 0.001; FE vs. CT: ^∗∗∗^*P* < 0.001; FE vs. ME: *P* > 0.05; *n* = 5 in each group. **(G)** Astrocyte differentiation in the DG of mice subjected to 6-week training. Quantification of the ratios of BrdU+GFAP+ cells to BrdU+ cells in the FE, ME, and CT groups. One-way ANOVA, *F*_2,12_ = 6.379, *P* = 0.013; *post hoc* test: ME vs. CT: ^∗^*P <* 0.05; ME vs. FE: *P* > 0.05; FE vs. CT: *P* > 0.05; *n* = 5 in each group.

At the end of training, mice subjected to different exercise paradigms were sacrificed and the Ki67 marker was used to evaluate cell proliferation in the DG (**Figure 2A**). To investigate cell survival in parallel, we pre-injected the same animals with an exogenous marker BrdU during the second week of treadmill running (**Figure 2B**). In line with numerous reports that running increases cell proliferation and neurogenesis in the adult DG (van Praag et al., 1999), the numbers of Ki67+ cells in CT, ME, and FE mice were 2743 ± 250, 4073 ± 242, and 3236 ± 148, respectively (*n* = 5 in each group; **Figure 2C**). The numbers of BrdU+ cells in the CT, ME, and FE groups were 1094 ± 146, 2500 ± 150, and 1483 ± 121, respectively (**Figure 2D**). These data suggest that ME but not FE increased the amount of proliferating (Ki67+) and surviving (BrdU+) cells. Nevertheless, it remained unclear whether the enhancement in cell survival was simply due to a higher rate of cell proliferation. To address this issue, we trained a new cohort of mice and injected them with BrdU during the second week of running as before. These animals were sacrificed 1 day after the last BrdU injection. Quantification of proliferating BrdU+ cells in the CT, ME and FE mice after 2-week running showed no significant difference between them (*n* = 5 in each group; **Figure 2E**), which was different from what we observed for quantification of surviving BrdU+ cells after 6-week running (**Figure 2D**). These data prompted us to speculate that exercise influenced NSC proliferation and the survival rate of newly generated cells separately. To exclude the possible influence of estrogens on hippocampal neurogenesis, we also counted BrdU+ cells in sedentary 14-week old females 1 day after BrdU+ injection. Similar to 6–7 week old mice (Lagace et al., 2007), these animals in different stages of the estrous cycle did not show significant differences in the number of proliferating cells (*n* = 5 in each group; **Supplementary Figure S1**), suggesting that the interference from variations in estrogen levels was minor.

To examine neuronal differentiation, we quantified the number of BrdU/NeuN double-labeled neurons in the same animals. The ratios of BrdU+NeuN+ cells to BrdU+ cells were 60.71 ± 2.35%, 87.54 ± 1.63%, and 84.53 ± 1.90%, respectively in the CT, ME, and FE groups, with no significant difference found between FE and ME mice (**Figure 2F**). We performed BrdU and GFAP double staining in a new cohort of mice. The percentages of GFAP-expressing cells among BrdU+ cells were 26.01 ± 5.18%, 7.01 ± 2.21%, and 18.98 ± 3.41%, respectively in the CT, ME, and FE groups, with no significant difference found between FE and ME mice (*n* = 5 in each group; **Figure 2G**). Taken together, our findings indicate that only ME upregulated both proliferation and differentiation of NSCs in the adult brain. However, both ME and FE may shift the balance between neurogenesis and gliogenesis in the adult hippocampus toward more production of neurons.

**FIGURE 3:**
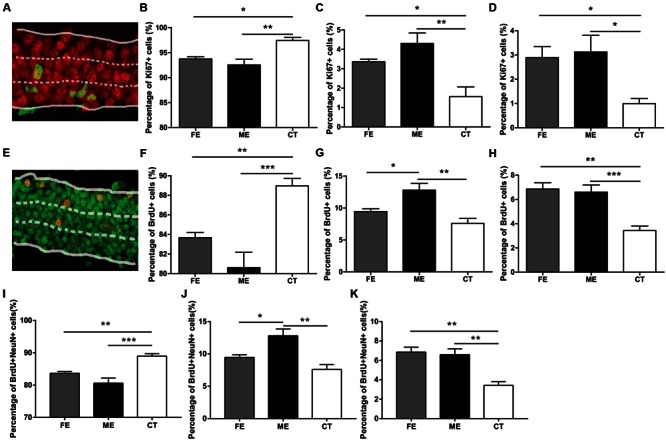
**Distribution of new neurons in the granule cell layer (GCL) of runners. (A)** A sample photomicrograph of Ki67+ cells in the GCL. **(B)** Both FE and ME groups have a lower percentage of Ki67+ cells located in the innermost GCL than the CT group. One-way ANOVA, *F*_2,12_ = 5.594, *P* = 0.0192; *post hoc* test: ME vs. CT: ^∗∗^*P <* 0.01; ME vs. FE: ^∗^*P* > 0.05; FE vs. CT: *P* > 0.05; *n* = 5 in each group. **(C)** Both FE and ME groups have a higher percentage of Ki67+ cells located in the middle GCL than the CT group. One-way ANOVA, *F*_2,12_ = 10.27, *P* = 0.0025; *post hoc* test: ME vs. CT: ^∗∗^*P <* 0.01; ME vs. FE: *P* > 0.05; FE vs. CT: ^∗^*P* < 0.05; *n* = 5 in each group. **(D)** Both FE and ME groups have a higher percentage of Ki67+ cells located in the outermost GCL than the CT group. One-way ANOVA, *F*_2,12_ = 10.80, *P* = 0.0021; *post hoc* test: ME vs. CT: ^∗^*P <* 0.05; ME vs. FE: *P* > 0.05; FE vs. CT: ^∗^*P* < 0.05; *n* = 5 in each group. **(E)** A sample photomicrograph of BrdU+ cells in the GCL. **(F)** Both FE and ME groups have a lower percentage of BrdU+ cells located in the innermost GCL than the CT group. One-way ANOVA, *F*_2,12_ = 14.29, *P* = 0.0007; *post hoc* test: ME vs. CT: ^∗∗∗^*P <* 0.001; ME vs. FE: *P* > 0.05; FE vs. CT: ^∗∗^*P* < 0.01; *n* = 5 in each group. **(G)** The ME group has a higher percentage of BrdU+ cells located in the middle GCL than the FE and CT groups. One-way ANOVA, *F*_2,12_ = 10.96, *P* = 0.0020; *post hoc* test: ME vs. CT: ^∗∗^*P <* 0.01; ME vs. FE: ^∗^*P* < 0.05; FE vs. CT: *P* > 0.05; *n* = 5 in each group. **(H)** Both FE and ME groups have a higher percentage of BrdU+ cells located in the outermost GCL than the CT group. One-way ANOVA, *F*_2,12_ = 16.25, *P* = 0.0004; *post hoc* test: ME vs. CT: ^∗∗∗^*P <* 0.001; ME vs. FE: *P* > 0.05; FE vs. CT: ^∗∗^*P* < 0.01; *n* = 5 in each group. **(I)** Both FE and ME groups have a lower percentage of BrdU+NeuN+ cells located in the innermost GCL than the CT group. One-way ANOVA, *F*_2,12_ = 16.25, *P* = 0.0004; *post hoc* test: ME vs. CT: ^∗∗∗^*P <* 0.001; ME vs. FE: *P* > 0.05; FE vs. CT: ^∗∗^*P* < 0.01; *n* = 5 in each group. **(J)** ME group has a higher percentage of BrdU+NeuN+ cells located in the middle GCL than the CT group. One-way ANOVA, *F*_2,12_ = 10.96, *P* = 0.0020; *post hoc* test: ME vs. CT: ^∗∗^*P <* 0.01; ME vs. FE: ^∗^*P* < 0.05; FE vs. CT: *P* > 0.05; *n* = 5 in each group. **(K)** Both FE and ME groups have a higher percentage of BrdU+NeuN+ cells located in the outermost GCL than the CT group. One-way ANOVA, *F*_2,12_ = 14.29, *P* = 0.0007; *post hoc* test: ME vs. CT: ^∗∗^*P <* 0.01; ME vs. FE: *P* > 0.05; FE vs. CT: ^∗∗^*P* < 0.01; *n* = 5 in each group.

In the adult DG, newly born neurons migrate a short distance radially into the GCL and a majority of them settles down in the inner one-third of the GCL (Mathews et al., 2010). In order to determine the correlation between exercise intensity and cell migration, we assessed the positions of newborn neurons in the DG of each group. When GCL was divided the into three layers of roughly identical thickness (**Figure 3A**), most of the Ki67+ cells were found in the innermost layer, no matter in CT, ME, or FE mice. Further quantification revealed that in CT mice, 97.45 ± 0.60% of Ki67+ cells resided in the inner one-third layer, whereas only 1.56 ± 0.50% and 1.0 ± 0.21% located in the middle and outer one-third layer, respectively. In contrast, 92.54 ± 1.13%, 4.30 ± 0.54%, and 3.12 ± 0.69% of Ki67+ cells were found in sequence from the innermost to outermost GCL in ME mice, while 93.74 ± 0.42%, 3.36 ± 0.12%, and 2.89 ± 0.46% were observed in FE mice (**Figures 3B–D**). It is likely that exercise enhances migration of NSCs. However, Ki67 shows universal expression in all proliferating cells and previous studies have revealed an increase in astrocyte proliferation after running (Li et al., 2005; Uda et al., 2006). We thus propose that exercise promotes division of cells located in the outer layers alternatively but not exclusively. The same analysis was applied to surviving BrdU+ cells from animals that had been exercised for 6 weeks and similar results were obtained (**Figures 3E–H**), except that fewer BrdU+ cells were found in the middle layer of FE mice than in that of ME mice. The distribution of BrdU+NeuN+ cells in each layer was very similar too (**Figures 3I–K**). Notably, proliferating BrdU+ cells from animals that had been exercised for 2 weeks did not exhibit any significant change in any layer (**Supplementary Figures S2A–C**). Together, these findings suggest that migration of newborn neurons was facilitated by forced running, but largely in an intensity-independent manner.

### Spatial Discrimination is Increased in ME but Not FE Mice

**FIGURE 4:**
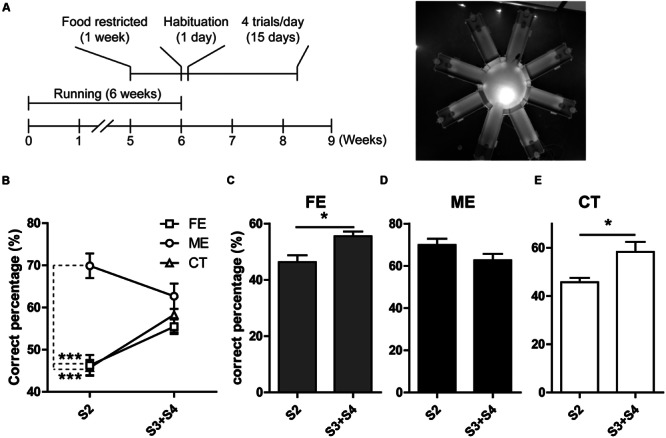
**Analysis of behavior in the RAM. (A)** Left, timeline of the RAM test. Right, photograph of the RAM arena. **(B)** ME but not FE increases the percent of correct choices at S2. Neither of them has effect on the percent of correct choices at S3 + S4 separations. Two-way ANOVA, *F*_2,18_ = 19.57, *P* < 0.0001; *post hoc* test: ME vs. CT: ^∗∗∗^*P* < 0.001; ME vs. FE: ^∗∗∗^*P* < 0.001; FE vs. CT: *P* > 0.05; *n* = 7 in each group. **(C)** The FE group shows a higher percent of correct choices at S3 + S4 than at S2. Paired *t*-test, ^∗^*P* < 0.05. **(D)** The ME group shows a percent of correct choices at S2 similar to that at S3 + S4. Paired *t*-test, *P >* 0.05. **(E)** The CT group has a higher percent of correct choices at S3 + S4 than at S2. Paired *t*-test, ^∗^*P* < 0.05.

Prior studies have revealed a positive correlation between the level of adult neurogenesis and the animal’s ability of pattern separation essential for the accuracy of memory encoding (Clelland et al., 2009). In the current study, we observed altered hippocampal neurogenesis in both FE and ME mice as compared to the CT group, and therefore assessed the potential effects on spatial discrimination utilizing a RAM testing paradigm (**Figure 4A**) as previously described (Clelland et al., 2009). In this task, mice were allowed to visit a sample arm and retrieve a food reward in this arm during the sample phase. During the choice phase, the animals were allowed to choose between the sample arm with no more food pellet and a new arm loaded with a reward. Mice were considered to make a correct choice only if they entered the new arm containing food reward. The spatial separation between these two arms varied from two to four arms. When the spatial separation was two arms, ME mice showed a significantly higher percent of making correct choice than FE and CT mice (**Figure 4B**). In contrast, when the spatial separation was three or four arms, there were no differences in the correct percent between all the groups (**Figure 4B**). Furthermore, neither FE nor CT mice could differentiate arms that were closely spaced (S2) as well as those that were more separated (S3 + S4), whereas the ME group did not show any significant change in their ability of spatial discrimination when the distance was decreased from S3 + S4 to S2 (**Figures 4C–E**). Thus, these results support the view that adult hippocampal neurogenesis is required for pattern separation, especially when the events are highly similar. Moreover, moderate but not high-intensity exercise enhanced behavioral spatial discrimination, although both training paradigms could improve neurogenesis in the adult DG. We noted a difference between Clelland’s study and ours: control mice in Clelland’s paper were able to distinguish well the closely located arms (S2), whereas the performance of our controls for the same pattern was close to chance, which may be accounted for by the difference in task difficulty. In Clelland’s paper, the wall of the maze was 2.5 cm in height, whereas the height of our maze wall was 23 cm. Thus, the visual cues were dramatically reduced for the animals in our tests.

### Intensity-Dependent Effects of Exercise on Neurotrophic Factors

**FIGURE 5:**
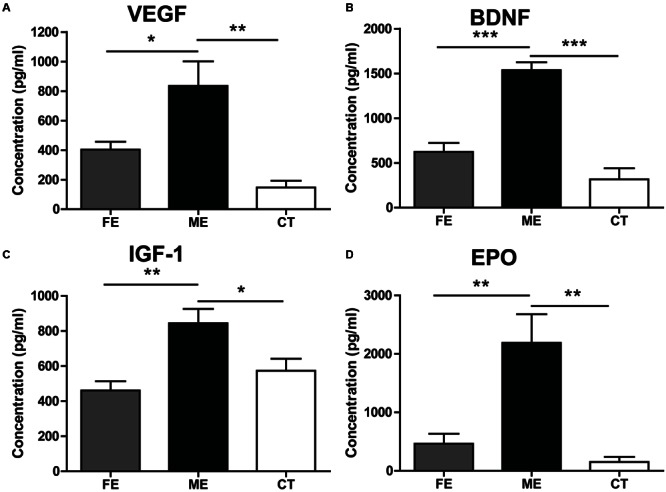
**Effects of running on expression of neurotrophic factors vary with intensity. (A)** Values of hippocampal VEGF levels. One-way ANOVA, *F*_2,12_ = 11.12, *P* = 0.0019; *post hoc* test: ME vs. CT: ^∗∗^*P* < 0.01; ME vs. FE: ^∗^*P* < 0.05; FE vs. CT: *P* > 0.05; *n* = 5 in each group. **(B)** Values of hippocampal brain-derived neurotrophic factor (BDNF) levels. One-way ANOVA, *F*_2,12_ = 36.43, *P* < 0.0001; *post hoc* test: ME vs. CT: ^∗∗∗^*P* < 0.001; ME vs. FE: ^∗∗∗^*P* < 0.001; FE vs. CT: *P* > 0.05; *n* = 5 in each group. **(C)** Values of IGF-1 levels in the brain. One-way ANOVA, *F*_2,12_ = 8.315, *P* = 0.0037; *post hoc* test: ME vs. CT: ^∗^*P* < 0.05; ME vs. FE: ^∗∗^*P* < 0.01; FE vs. CT: *P* > 0.05; *n* = 5 in each group. **(D)** Values of hippocampal EPO levels. One-way ANOVA, *F*_2,12_ = 13.14, *P* < 0.0009; *post hoc* test: ME vs. CT: ^∗∗^*P* < 0.01; ME vs. FE: ^∗∗^*P* < 0.01; FE vs. CT: *P* > 0.05; *n* = 5 in each group.

Adult neurogenesis can be regulated by neurotrophic factors involved in a broad spectrum of physiological changes in the brain (Mu et al., 2010). Here, we investigated the protein levels of VEGF, BDNF, IGF-1, and EPO to explore potential molecular indicators of exercise intensity and their association with hippocampal neurogenesis. VEGF expression in the hippocampus was found to increase fivefold in the ME group compared with the CT group (**Figure 5A**). VEGF contents were not significantly different between the FE and CT groups. Similarly, hippocampal BDNF levels in FE mice were apparently lower than those in ME mice, while no significant difference was found between the FE and CT groups (**Figure 5B**). The IGF-1 amounts in brain tissue of FE mice were about the same level as those in the CT group, but significantly lower than those in the ME group (**Figure 5C**). Especially, very low concentrations of hippocampal EPO were detected in CT mice and its dramatic elevation was found in the ME but not FE group (**Figure 5D**). Thus, these results indicate that changes of VEGF, BDNF, IGF-1, and EPO concentrations were all inversely correlated with the intensity of exercise. In other words, ME could enhance the level of neurotrophic factors including VEGF, BDNF, IGF-1, and EPO, whereas intense exercise could not.

### Exercise-Induced Mitochondrial Changes

**FIGURE 6:**
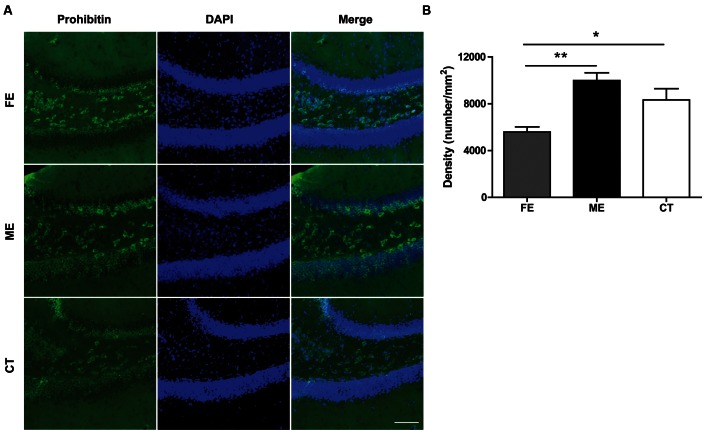
**Prohibitin is downregulated in FE mice. (A)** Representative images of prohibitin immunoreactivity in the DG regions of FE, ME and CT mice. Scale bar = 100 μm. **(B)** Quantification of prohibitin+ structures in each group. One-way ANOVA, *F*_2,12_ = 9.668, *P* < 0.0032; *post hoc* test: ME vs. CT: *P* > 0.05; ME vs. FE: *P* < 0.01; FE vs. CT: *P* > 0.05; *n* = 5 in each group.

Regular physical exercise has been reported to augment brain mitochondrial activity (Bayod et al., 2011). Since prohibitin is a protein expressed prevalently in mitochondria and exerts neuroprotective effect by preserving mitochondrial function (Artal-Sanz and Tavernarakis, 2009), here we surveyed the presence of mitochondria in the DG utilizing prohibitin as the marker. As shown in **Figure 6A**, a higher prohibitin immunoreactivity in the DG was found in the ME group than in the FE group. A further quantification revealed that FE induced a significant decrease in the number of prohibitin+ cells compared to ME or CT mice (**Figure 6B**). Thus, chronic FE in this study altered the mitochondrial function in the DG.

## Discussion

It has been reported that low- to moderate- but not high-intensity intensity exercise can enhance adult hippocampal neurogenesis; however, exercise of high-intensity is usually associated with significant more production of stress hormones in those studies (Inoue et al., 2015). To exclude the interference from stress, we replaced the commonly used electric shock with a gentle tail tapping in forced treadmill running, which led to similar levels of serum cortisol in FE and ME mice, and therefore allowed us to compare the effects of moderate- and high-intensity training alone. Here, we observed that ME enhanced cell proliferation, survival, neuronal differentiation, and migration, whereas FE only promoted neuronal differentiation and migration. This phenomenon was accompanied with a reduction in mitochondrial activity and lower expressions of VEGF, BDNF, IGF-1, and EPO in the FE group than in the ME group.

To assess the function of enhanced adult hippocampal neurogenesis induced by physical exercise, we tested whether FE and ME mice could differentiate between locations that were closely spaced versus those that were highly separated. Consistent with previous reports (Clelland et al., 2009; Sahay et al., 2011), ME improved the animals’ ability to distinguish locations with an S2 separation (**Figure 4B**). However, FE did not have the same effect on spatial discrimination although it enhanced differentiation of NSCs to neurons (**Figure 2F**). One possible reason is that the lack of parallel increase in some key factors (e.g., VEGF, BDNF, IGF-1, and/or EPO) in FE mice compared to ME mice (**Figure 5**) may influence the maturation and physiological properties of newborn neurons, which finally make them less contributable to pattern separation.

Previous studies have shown that physical exercise can upregulate a variety of neurotrophic factors. Among these, BDNF has been intensively studied and shown to be involved in modulation of neurogenesis by exercise (Vivar et al., 2013). Given these findings, we expected that ME could increase hippocampal BDNF. The result in **Figure 5B**, showing much higher level of BDNF in the ME group than in the CT group, did confirm this effect. In contrast, there was no significant difference in BDNF amounts between the FE and CT groups, indicating that FE could not increase hippocampal BDNF. Despite the putative role of BDNF in neurogenesis, there is still debate in regard to its participation in specific aspects of this process. Specifically, conflicting results have been reported about the impact of BDNF on cell proliferation, differentiation and survival in the adult DG (Ahmed et al., 1995; Sairanen et al., 2005). Our present study shows that hippocampal BDNF levels were significantly reduced in FE mice as compared with ME mice. Since the FE group had significantly fewer Ki67+ and BrdU+ cells than the ME group, whereas the ratios of BrdU+NeuN+ cells to BrdU+ cells were similar in these two groups (**Figure 2**), these observations support a role for BDNF in the proliferation of progenitors and the survival of new neurons, but not in neuronal differentiation. In **Figure 5C**, similar results have been found for IGF-1, another important determinant of exercise-induced changes in the adult DG (Trejo et al., 2001). In fact, some evidence indicates that there are points of convergence between IGF-1 and BDNF signaling (Ding et al., 2006). For instance, IGF-1 increases BDNF signaling by upregulating levels of TrkB. Blocking IGF-1 signaling prevents exercise-dependent elevation of hippocampal BDNF and attenuates the induction of synaptic proteins downstream from TrkB signaling (Ding et al., 2006). Consistent with these reports, here we observed that both BDNF and IGF-1 were increased by ME, while neither was increased by FE.

Vascular endothelial growth factor is also required for the effects of running on adult hippocampal neurogenesis (Fabel et al., 2003). On the one hand, VEGF can promote neurogenesis indirectly by stimulating endothelial cell production and the release of other relevant neurotrophic factors such as BDNF (Louissaint et al., 2002). On the other hand, it can directly stimulate division of neural precursor cells through Flk-1-mediated signaling (Cao et al., 2004). Notably, VEGF expression is increased during hypoxic exposure and inhibition of VEGF signaling impedes brain recovery after stroke or traumatic brain injury (Schratzberger et al., 2000). Similar to VEGF, EPO is produced under hypoxic conditions, which can protect neurons from oxidative stress, ischemia, and stroke (Ghezzi and Brines, 2004). EPO also stimulates proliferation and prevents apoptosis by promoting oxygen delivery to the brain or by direct interaction with neural progenitor cells (Shingo et al., 2001; Chen et al., 2007). These hypoxia-inducible neuroprotective proteins are particularly relevant to the impact of running on the brain, given that exercise may lead to diminished cerebral blood flow and therefore reduced oxygen supply and delivery to the brain (Sato et al., 2011). Indeed, upregulation of both VEGF and EPO was observed in the hippocampus after chronic training, although their increase in FE mice did not reach a significance level (**Figures 5A,D**). Surprisingly, EPO amount in the FE group was lower than in the ME group, as the concentration of EPO is presumably inversely related to the oxygen content of the blood. We speculate that this is probably due to the adaption of FE mice to a more hypoxic environment and consequently a less vigorous response to further hypoxic challenge. Maybe for a similar reason, prohibitin, an essential mitochondrial protein that is upregulated by transient ischemia or oxygen-glucose deprivation (Zhou et al., 2012; Kurinami et al., 2014), showed a less elevation in the FE group than in the ME group (**Figure 6**).

Overall, our findings suggest that intense exercise can still enhance neuronal differentiation and migration. However, the neurogenic effects of exercise are in general inversely related to its intensity. Correspondingly, moderate but not intense exercise can improve spatial memory performance. Although our study suggests a positive correlation between specific aspects of neurogenesis and the expression of neurotrophic factors in exercised mice, it is not known whether these factors are causally linked to enhanced neurogenesis. A further study is required to clarify this issue. The results may help to optimize therapeutic strategies aiming at restoring hippocampal functions in the case of brain injury or disorders.

## Author Contributions

Study design: YM and JS. Performance of experiments: JS, CH, MG, GC, and LZ. Data analysis: JS, CH, MG, and YL. Manuscript writing: YM and YL.

## Conflict of Interest Statement

The authors declare that the research was conducted in the absence of any commercial or financial relationships that could be construed as a potential conflict of interest.

## Supplementary Material

FIGURE S1 **The number of proliferating cells was not changed throughout the estrous cycle in sedentary 14-week-old females.** No significant difference in the number of BrdU+ cells was detected in different stages of the estrous cycle. One-way ANOVA, *F*_2,12_ = 0.1776, *P* = 0.8395; *n* = 5 in each group.

FIGURE S2 **Distribution of nascent cells in the GCL of 2- or 6-week runners. (A)** No significant difference between all groups in the number of innermost GCL BrdU+ cells. One-way ANOVA, *F*_2,12_ = 0.074, *P* = 0.929; *post hoc* test: ME vs. CT: *P* > 0.05; ME vs. FE: *P* > 0.05; FE vs. CT: *P* > 0.05; *n* = 5 in each group. **(B)** No significant difference between all groups in the number of middle GCL BrdU+ cells. One-way ANOVA, *F*_2,12_ = 0.71, *P* = 0.51; *post hoc* test: ME vs. CT: *P* > 0.05; ME vs. FE: *P* > 0.05; FE vs. CT: *P* > 0.05; *n* = 5 in each group. **(C)** No significant difference between all groups in the number of outermost GCL BrdU+ cells. One-way ANOVA, *F*_2,12_ = 2.018, *P* = 0.17; *post hoc* test: ME vs. CT: *P* > 0.05; ME vs. FE: *P* > 0.05; FE vs. CT: *P* > 0.05; *n* = 5 in each group.
