# Corrigendum: Intense Exercise Promotes Adult Hippocampal Neurogenesis But Not Spatial Discrimination

**DOI:** 10.3389/fncel.2019.00303

**Published:** 2019-07-09

**Authors:** Ji H. So, Chao Huang, Minyan Ge, Guangyao Cai, Lanqiu Zhang, Yisheng Lu, Yangling Mu

**Affiliations:** ^1^Department of Physiology, School of Basic Medicine, Huazhong University of Science and Technology, Wuhan, China; ^2^Tianjin Institute of Integrative Medicine for Acute Abdominal Disease, Nankai Hospital, Tianjin, China; ^3^Institute of Brain Research, Collaborative Innovation Center for Brain Science, Huazhong University of Science and Technology, Wuhan, China; ^4^Hubei Key Laboratory of Drug Target Research and Pharmacodynamic Evaluation, Huazhong University of Science and Technology, Wuhan, China

**Keywords:** hippocampus, pattern separation, neurotrophic factors, erythropoietin, prohibitin

The equal contribution footnote was missing for author Chao Huang. Chao Huang was one of the co-first author which had contributed equally to this work. The original article has been updated.

