# Supplementary figures and images for: Intense Exercise Promotes Adult Hippocampal Neurogenesis But Not Spatial Discrimination
Source: Front Cell Neurosci. 2017 Jan 31;11:13. doi: 10.3389/fncel.2017.00013 (PMC5281566; doi:10.3389/fncel.2017.00013)

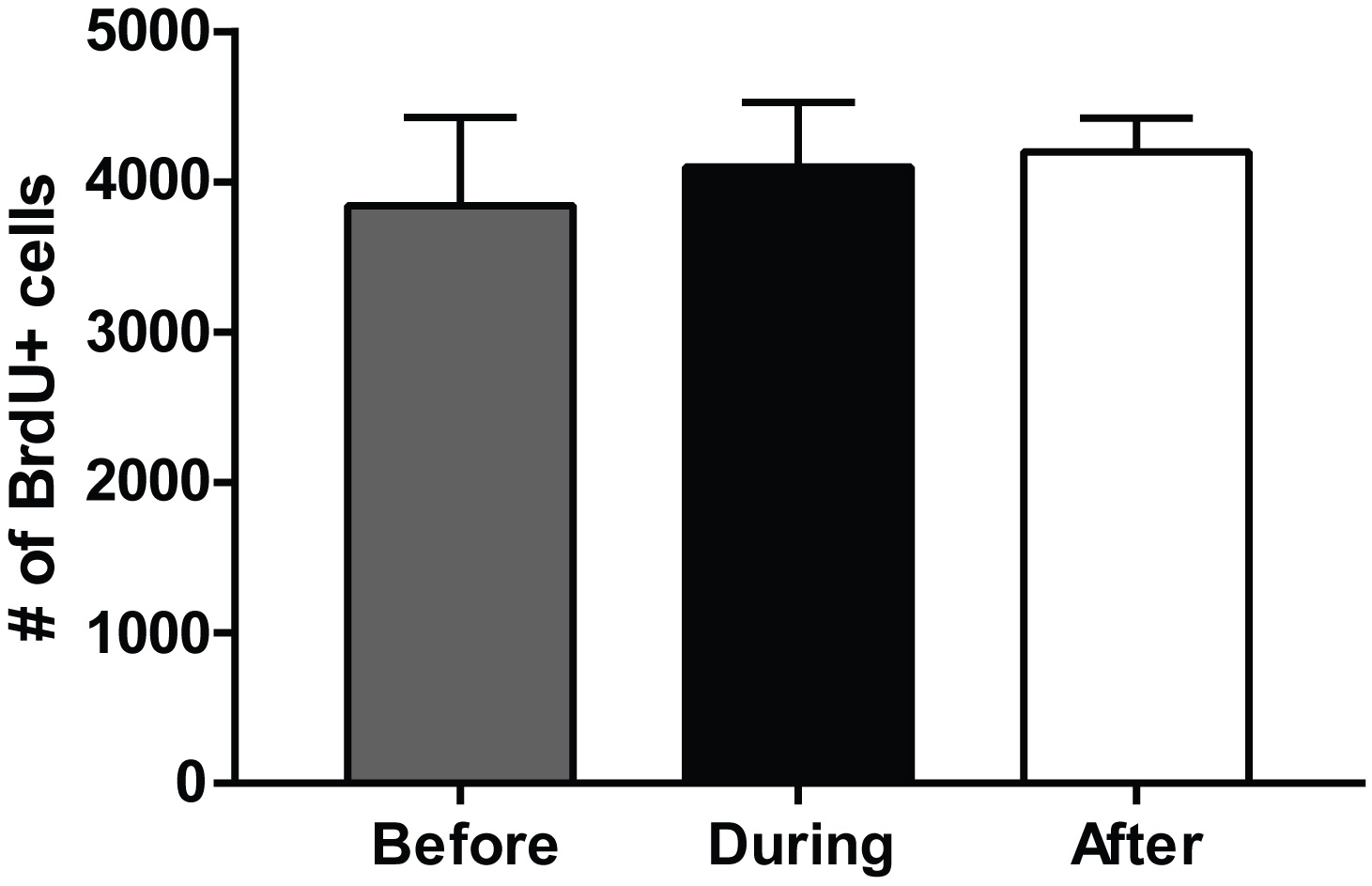

Supplement: FIGURE S1 — The number of proliferating cells was not changed throughout the estrous cycle in sedentary 14-week-old females. No significant difference in the number of BrdU+ cells was detected in different stages of the estrous cycle. One-way ANOVA, F2,12 = 0.1776, P = 0.8395; n = 5 in each group. [file Image_1.JPEG]

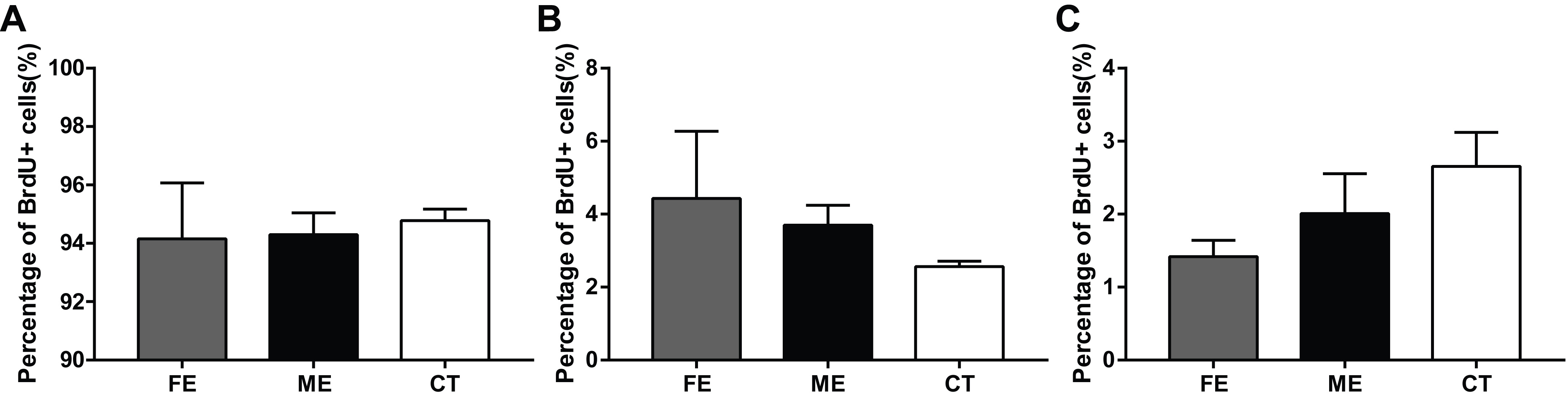

Supplement: FIGURE S2 — Distribution of nascent cells in the GCL of 2- or 6-week runners. (A) No significant difference between all groups in the number of innermost GCL BrdU+ cells. One-way ANOVA, F2,12 = 0.074, P = 0.929; post hoc test: ME vs. CT: P > 0.05; ME vs. FE: P > 0.05; FE vs. CT: P > 0.05; n = 5 in each group. (B) No significant difference between all groups in the number of middle GCL BrdU+ cells. One-way ANOVA, F2,12 = 0.71, P = 0.51; post hoc test: ME vs. CT: P > 0.05; ME vs. FE: P > 0.05; FE vs. CT: P > 0.05; n = 5 in each group. (C) No significant difference between all groups in the number of outermost GCL BrdU+ cells. One-way ANOVA, F2,12 = 2.018, P = 0.17; post hoc test: ME vs. CT: P > 0.05; ME vs. FE: P > 0.05; FE vs. CT: P > 0.05; n = 5 in each group. [file Image_2.JPEG]
